# Implementing a co-designed communication training programme for non-Indigenous mental health professionals working with Aboriginal and/or Torres Strait Islander consumers in Queensland, Australia: protocol

**DOI:** 10.1136/bmjopen-2026-117447

**Published:** 2026-10-29

**Authors:** Edwin Adrianta Surijah, Christina M Bernardes, Stuart Ekberg, Julie-Anne Rogers, Jean Marie Spinks, Renata Meuter, Kylie Burke, Gabrielle Paton Simcock, Michelle Combo, Ilana Mushin, Greg Pratt

**Affiliations:** 1 Jawun Research Institute, Central Queensland University, Brisbane, Queensland, Australia; 2 The University of Queensland, Brisbane, Queensland, Australia; 3 Institute for Urban Indigenous Health Ltd, Windsor, Queensland, Australia; 4 Flinders University, Adelaide, South Australia, Australia; 5 Centre for Applied Health Economics, Menzies Institute for Health Queensland, The University of Queensland, Saint Lucia, Queensland, Australia; 6 School of Psychology and Counselling, Queensland University of Technology, Brisbane, Queensland, Australia; 7 The University of Queensland School of Psychology, Saint Lucia, Queensland, Australia; 8 ARC Centre of Excellence for Children and Families over the Life Course, Indooroopilly, Queensland, Australia; 9 Gnibi College of Indigenous Australian Peoples, Southern Cross University, Lismore, New South Wales, Australia; 10 School of Languages and Cultures, The University of Queensland, Brisbane, Queensland, Australia

**Keywords:** MENTAL HEALTH, Health Services, Delivery of Health Care, Integrated, Community Participation

## Abstract

**Abstract:**

**Introduction:**

This protocol outlines co-design processes used in the development of communication training for non-Indigenous mental health professionals working in the public hospital and health service sector in Queensland, Australia, alongside governance procedures used to ensure that Indigenous research methods are applied appropriately. Intercultural communication between Aboriginal and/or Torres Strait Islander consumers and non-Indigenous health professionals can adversely affect the delivery of health services. In a mental health service setting, communication can negatively impact the therapeutic relationship between health providers and service users, alongside treatment outcomes for these people. This training programme aims to enhance the communication skills of non-Indigenous mental health professionals and improve healthcare provided to Aboriginal and/or Torres Strait Islander consumers.

**Methods:**

This is a mixed-methods, co-designed single-arm interventional study (communication training programme) to be implemented in a staggered manner, complemented by a matched-sample observational study. The communication training will be delivered to non-Indigenous mental health professionals within Queensland Health. The voices, leadership and perspectives of Aboriginal and Torres Strait Islander peoples serve as the principal guiding element and key component of the communication training programme. They will be actively involved as members of the research team, steering committee, working groups and/or advisory groups throughout the study.

**Ethics and dissemination:**

The ethical considerations for this study are primarily guided by the Australian Institute of Aboriginal and Torres Strait Islander Studies (AIATSIS) Code of Ethics for Aboriginal and Torres Strait Islander Research, which addresses issues such as data sovereignty, data storage and risk mitigation. The Principal Investigator and the Steering Committee, which comprises Indigenous members of the research team, made decisions regarding how the data are stored and analysed. They also determined how the study findings are published and returned to participating services, advisory groups, and Aboriginal and Torres Strait Islander communities, while ensuring that all publications accurately reflect the experiences and knowledges of Indigenous peoples. The study is also being conducted in collaboration with Queensland Health, which will provide support for the management of participant distress. Procedures to manage participant distress include the right to pause or withdraw from the study at any time, as well as access to appropriate support pathways should distress arise.

**STRENGTHS AND LIMITATIONS OF THIS STUDY**

- This study is informed by, and co-designed with, multiple stakeholders; most importantly, Aboriginal and Torres Strait Islander peoples from different regions in Queensland.
- The research governance includes a steering committee, working groups and advisory groups to ensure the study is conducted in a culturally sensitive manner.
- The study will implement a culturally sensitive data-gathering approach known as yarning.
- To ensure maximum participation of mental health professionals and benefits for consumers, the study design must use a single-arm interventional approach rather than a randomised controlled trial.

## Introduction

 Effective communication between clinicians and First Nations patients/consumers underpins the delivery of high-quality, culturally safe and equitable healthcare.^1 2^ Beyond the ongoing impacts of colonisation experienced by First Nations peoples, effective communication is particularly critical in mental healthcare, where clinician–patient communication serves as a primary diagnostic and therapeutic tool.^3^ Communication barriers are also recognised as major contributors to disparities in mental health outcomes among First Nations consumers.^2^

This protocol describes the co-design processes used to develop a communication training programme for non-Indigenous mental health professionals working in the public mental health service sector in Queensland, Australia. It highlights the importance of embedding Aboriginal and Torres Strait Islander governance and research methods (eg, yarning circles), at the core of research and implementation science. The National Aboriginal and Torres Strait Islander Leadership in Mental Health emphasised the need for culturally appropriate and accountable mental health services.^4^ Throughout this document, the terms ‘Indigenous’, ‘First Nations’ and ‘Aboriginal and/or Torres Strait Islander’ are used interchangeably. We acknowledge that the use of these terms is a consequence of an aim to support readability of this document and recognise the limitations of these terms in paying respect to the multitude of diverse and unique Indigenous peoples of the continent, ‘Australia’. We acknowledge the rich traditional, cultural, social and spiritual diversity of the First peoples of Australia and their sovereign rights and responsibilities as the custodians and caretakers of the lands, air and waters with whom they share an ancient connection. For Indigenous peoples, culture is a central and significant factor that influences and impacts health and well-being of not only an individual but their family, community, connection to place and spirit.^5^ Within a mainstream public mental health service setting, healthcare is often provided by non-Indigenous health professionals.^6^

Addressing the burden of mental ill-health among Indigenous Australians is a national priority.^7 8^ The impact of economic and social disadvantage and trauma (as a consequence of discrimination, colonisation and inequitable access to employment, education and housing) on the mental health of Aboriginal and Torres Strait Islander people is confronting and consistently highlighted.^8 9^ Government reports indicate increased levels of unmet need, resource scarcity and significant disparities.^8 10^ Moreover, according to the Australian Institute of Health and Welfare,^10^ mental ill-health and substance use disorders are the leading causes of disease and injury burden for Aboriginal and/or Torres Strait Islander people; specifically, mood and anxiety disorders, schizophrenia and alcohol and other drug use disorders. Estimates of the prevalence of mental ill-health experienced by Aboriginal and Torres Strait Islander people in Queensland describe that anxiety and depression are particularly significant and vary based on relative geographic (ie, remoteness) access to services.^11^

Given the data mentioned before, services need to be sensitive to the realities of everyday life for Aboriginal and Torres Strait Islander Australians.^12^ However, the intercultural interaction that occurs when a non-Indigenous health professional provides care to an Aboriginal and/or Torres Strait Islander consumer can create communication gaps that affect the quality of health services. More importantly, in a mental health service setting, compassionate and competent intercultural interactions are exceedingly important to delivering high-quality mental healthcare.^2^

Provided that, the health service sector needs support to understand how the delivery of services influences the experience of quality healthcare, and how these inform culturally safe practice.^13^ Supporting staff to practice respectful, reciprocal, and person-, family- and community-centric communication is crucial to ensuring that health services are culturally sensitive and safe.^4^ Ultimately, healthcare services that recognise culture as a source of strength, rectify assumptions and negative perceptions, demonstrate respect for Indigenous ways of knowing, being and doing, and that make an investment in relationships built on respect, serve as the gold standard for quality healthcare.^14^

Understanding what constitutes culturally appropriate communication with Aboriginal and Torres Strait Islander consumers requires the use of culturally appropriate co-design methodology (eg, yarning).^15^ Indigenous research methods provide culturally safe and relational ways of gathering knowledge that may not be accessible through conventional data collection approaches. By generating evidence that is grounded in Indigenous worldviews and lived experiences, these methods can better inform service design, delivery, and quality improvement initiatives, contributing to meaningful service-level change and improved outcomes. Yarning is more than a conversational data collection technique; it is a culturally grounded process that builds trust and respectful relationships through shared dialogue, active listening and the recognition of participants as knowledge holders. The yarning process allows participants to share experiences, stories and reflections in ways that are culturally safe and meaningful, enabling knowledge to emerge collaboratively rather than being extracted by researchers.

Previous studies have shown the positive effects of co-designed communication training for non-Indigenous clinicians on other aspects of Indigenous healthcare.^16–19^ The protocol presented here describes the co-design, implementation and evaluation of a communication training programme to improve the communication skills of non-Indigenous mental health professionals working with Indigenous consumers to empower clinicians to reflect on how their ways of thinking, being, and doing influence their worldview of the mental health and well-being of Indigenous peoples. Given the scope of the study, this protocol is divided into two phases. Phase 1 focuses on the co-design process of the training programme, while Phase 2 focuses on its delivery to non-Indigenous mental health professionals. We also set specific aims for each phase as follows:

### Aims (phase 1) are to

(A1): Establish Indigenous governance that supports leadership through majority membership.

(A2): Identify the communication needs and preferences of Indigenous consumers of mental health services.

(A3): Identify the communication training needs of mental health professionals providing care to Indigenous peoples.

(A4): Co-design with Indigenous consumers and health clinicians a culturally sensitive communication training experience for non-Indigenous mental health clinicians.

### Aims (phase 2) are to

(A5): Evaluate the effectiveness of a communication training programme for non-Indigenous mental health professionals across four sites in enhancing the skills, knowledge and attitudes of mental health professionals regarding communication with Indigenous consumers.

(A6): Evaluate the efficacy of the communication training programme for non-Indigenous mental health professionals in enhancing health outcomes and satisfaction among Indigenous people receiving mental healthcare from public mental health service providers.

## Methods

### Study design and setting

This is a mixed-methods, co-designed, single-arm interventional study evaluating a communication training programme for mental health clinicians. The study comprises two phases (figure 1). Phase 1 will focus on the engagement and co-design of the communication training package (CTP) with mental health professionals and consumers. Phase 2 will involve the implementation and evaluation of the training programme. The evaluation employs a single-arm, non-randomised pre–post intervention design, with the training programme rolled out sequentially across participating services in a staggered manner.^20^

**Figure 1:**
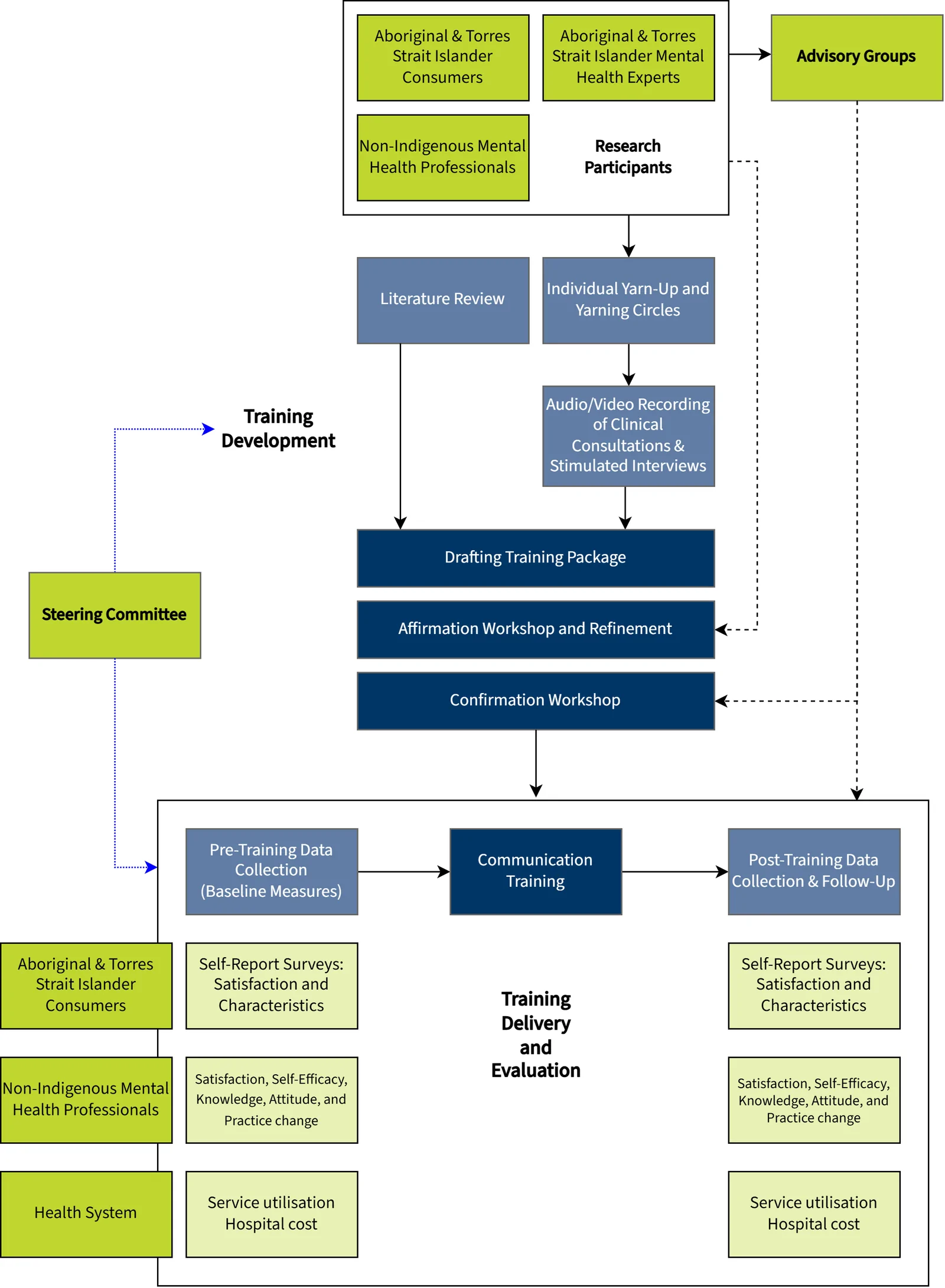
Co-design, implementation, and evaluation of the communication training programme for non-Indigenous mental health professionals.

Outcomes will be assessed before and after training to examine changes associated with the intervention. To complement the primary pre–post evaluation, matched-sample observations derived from clinical health records will be analysed to provide additional evidence regarding the impact of the training programme. The matched-sample study refers to data requested from medical records, in which consumers’ data will be matched with those of their treating mental health professionals. Changes in consumers’ service access rates, health outcomes and other relevant indices will be examined before and after the communication training attended by mental health professionals. These observational analyses serve as a supplementary component of the evaluation rather than a separate primary study design.

### Patient and public involvement

Figure 1 outlines the proposed involvement of research participants throughout the study cycle. Indigenous patients and consumers of mental health services will be involved in the study to share their experiences of interacting with mental health professionals and to participate as members of an advisory group, informing the development of the CTP. Aboriginal and Torres Strait Islander mental health experts and non-Indigenous mental health professionals will also be invited to join separate advisory groups.

The research team will engage these advisory groups during the confirmation workshop to present the finalised draft of the CTP, ensuring that the training is culturally sensitive and that its content aligns with the aims of the study. The non-Indigenous mental health professional advisory group will also provide input on the delivery of the training, including the preferred timing and location of the sessions, as well as the time required to complete the survey. In addition, all research participants will receive annual newsletters to keep them informed of the study’s progress.

### Phase 1

Phase 1 will employ a participatory action research framework^21^ to co-design a communication training programme. We acknowledge the vast geographical context of Australia; therefore, in this study, we specifically collaborated with Queensland Health in Queensland, Australia, to narrow the setting for the co-design and subsequent training delivery. The study setting involves four large Hospital and Health Services (HHSs) in Queensland, Australia: Metro North, Metro South, Central Queensland and Cairns and Hinterland. HHSs are funded by the state health department to provide healthcare services to the public in their geographic catchment. Specialist mental health, alcohol and other drug (MHAOD) inpatient and community-based assessment, treatment, rehabilitation and recovery services comprise a core function within HHSs. These MHAOD specialist services provide care to people experiencing moderate to severe and complex mental ill-health (eg, mood disorders, psychosis, schizophrenia).

A distinct feature of this study is its co-design approach, which ensures that the voices of Aboriginal and/or Torres Strait Islander peoples are prioritised and privileged throughout the research process. Indigenous peoples were involved in all aspects of the study, serving as both leaders and participants as chief and associate investigators. In addition, Indigenous peoples who also have a lived and living experience of mental ill-health serve on the Steering Committee, participate in the advisory groups and contribute to and inform the development of the training as valued partners. This is illustrated in figure 1.

Phase 1 has been partially completed, with the yarning circles involving non-Indigenous mental health professionals and Aboriginal and Torres Strait Islander mental health workers conducted between 18 November 2022 and 8 November 2024. Individual yarn-ups with consumers commenced on 16 June 2025 and are still ongoing. The recording of clinical consultations is planned for 2026.

#### Participants and sample size

Three groups of participants will be involved in the study, with the following inclusion criteria:

Non-Indigenous mental health professionals (eg, nurses, doctors and allied health clinicians) who provide healthcare services to Indigenous peoples experiencing mental ill-health.
Consumers: Adult (18+years) Indigenous peoples with lived experiences of mental health issues receiving care in the participating hospitals and health services and/or family members, carers and/or support people (with consent of the person with the lived experience of mental ill-health). The term ‘consumers’ is used to refer to people with a lived experience of mental ill-health and/or supporting people with a lived experience of mental ill-health.
(3) Mental health experts: This group refers to Indigenous health workforce, academia, researchers and community members with valued experience or expertise in mental health and ill-health.

Exclusion criteria are as follows:

1. If a person is unable or unwilling to provide informed consent.
2. If a person with lived experiences of mental health issues has a significant cognitive or severe intellectual impairment or is experiencing an exacerbation of symptoms that align with the person experiencing an acute episode of mental illness and that compromises their ability to make informed decisions and provide consent.
3. If a person being offered the opportunity to participate is unable to communicate in English and where an interpreter is not available.
4. In the case of inviting a family member, carer and/or support person to participate, where a person with a lived experience of mental ill-health does not consent to their family member, carer or support person being contacted and invited to participate in the study.

Each HHS site comprises numerous services, for instance, community mental health services or hospitals. At least one yarning circle will be facilitated for each site and participant group; the nominal number of participants per circle will be between 5 and 15.

#### Recruitment

The recruitment process will be the same for both phases of the study. Study site coordinators will engage with the leadership group and individual staff for each relevant mental health service at each HHS study site. By invitation, the research team will attend meetings with service staff to raise awareness of the study and the opportunity to participate. Nominations of eligible consumers will be sought from mental health professionals of local services. The research team will contact nominated consumers to provide information about the study, gauge interest and seek consent to participate. Mental health professionals will be invited to participate in the study via an email from the research team. The opportunity to participate in the study will be promoted at mental health service team meetings and via emails.

#### Procedures

##### Study governance

The leadership of Indigenous peoples plays a leading role throughout the study, co-design and conduct. This emphasis is reflected in the Steering Committee, a governance entity comprising researchers and mental health professionals, the majority of whom are Aboriginal and/or Torres Strait Islander. In addition, three advisory groups will be established: a Consumer Advisory Group (CAG), a Health Professionals Advisory Group (HPAG) and a Mental Health Expert Advisory Group.

Acceptability and validity of the communication training programme will be evaluated by the HPAG. Advisory group members will also evaluate the survey tools to be used in Phase 2, including the baseline and follow-up assessments for health professionals (see table 1). This assessment of the communication training programme and its associated survey tools by the HPAG will use a four-point Likert scale (1: not relevant, 2: needs revision, 3: relevant but needs minor revision and 4: very relevant).

**Table 1:** Outcomes and measures to evaluate the communication training programme

Domains	Source of data	t0	t1	f	Outcome level
Non-Indigenous mental health professional-level					
Satisfaction and Acceptability	Training Acceptability Rating Scale TRAS-2 (^30^; Milne & Noone, 1996)		✔		Secondary
Self-efficacy	Self-Efficacy Questionnaire (SE-12)^31^	✔	✔		Secondary
Knowledge	To be developed once the training content has been fully co-designed	✔	✔		Primary
Attitude	Attitude Towards Medical Communication Scale^32^	✔	✔	✔	Secondary
Practice change	Observational analysis (audio/video recording and clinical notes)	✔	✔	✔	Primary
Consumer-level					
Experience	Self-reported survey: Patient-reported Experience^7^	✔		✔	Secondary
Characteristics	Kim Alliance Scale^36^	✔		✔	Secondary
Health system-level					
Service utilisation	Consumer Integrated Mental Health and Addiction (CIMHA)	✔		✔	Secondary
Cost-effectiveness	National Hospital Cost Data Collection	✔		✔	Secondary
Socio-demographic covariates					
Socio-demographics	Age, education level, length of experience working in the field and occupational status	✔	✔	✔	Covariate

f, follow-up one month and six months after training/intervention; t0, baseline; t1, post-intervention.

##### Development of the communication training programme

Yarning, as an accepted Indigenous research method for qualitative data collection method,^15 22^ will be used in Phase 1 to gather information necessary to identify core content of the training programme, confirmation of preferred training medium and modes for use in the delivery of the training. In this study, the working definition for yarning or yarning circles is a process of relationship building and information sharing for the creation of new knowledge.^23^ Relationship building will occur through the establishment of culturally safe spaces, which use appropriate introduction and informal conversations, the recognition of local cultural protocols (e.g. Acknowledgement of Country, Elders, key staff) and the facilitation of discussions by researchers experienced in yarning. Participants will discuss issues regarding communication within mental health services, allowing priorities and insights to emerge organically. This enables the generation of new knowledge, creating opportunities for reflection and identification of shared strengths and solutions. At each study site, yarning will be adapted to local customs and community expectations.

Guidance from local Aboriginal and Torres Strait Islander mental health professionals will inform decisions regarding participant recruitment, the composition of yarning groups, appropriate venues, facilitation processes and the management of culturally sensitive topics. This flexibility recognises the diversity of Aboriginal and Torres Strait Islander communities and ensures that the research process remains locally relevant, respectful and culturally responsive. The yarning circles will also explore mental health professionals’ preferences for the communication training programme (eg, duration, mode of delivery, training resources). Findings from the yarning process will inform the development of a draft communication training programme, which will subsequently be subjected to iterative cycles of affirmation, refinement and confirmation workshops in conjunction with the participants who wish to join the Consumer Advisory Group in Phase 2 of the study.

Observation of interactions between consumers and mental health professionals (video-recordings of clinical interactions), yarning circles with consumers and health providers and/or individual yarn-ups across each of the four study sites support identification of communication needs and preferences, and the form and function of the training. Service policies, documents and video-recorded materials available in the public domain will be incorporated into the analysis if deemed appropriate. Recordings of clinical interactions will be captured to accurately represent the mental health professional‒consumer communication.

These information sources and resources will help in identifying practices that result in what consumers and health professionals consider ‘successful and culturally competent interactions’. The focus on positive interaction enables us to develop a strengths-based analytic foundation that will be used to draft an initial communication training programme. The yarning circles also explore mental health professionals’ preferences for the communication training programme, such as duration, mode of delivery and educational resources to be used in the training. The draft of the communication training programme will be submitted to cycles of affirmation, refinement and confirmation workshops.

### Data analysis

Qualitative data generated through yarning circles and individual yarn-ups will be audio-recorded and transcribed verbatim before being thematically analysed using the reflexive thematic analysis method^24^ to identify categories of needs and preferences that will be incorporated into the communication training programme. The analysis will be co-conducted with the Indigenous researcher to ensure the inclusion of the Indigenous cultural lens and worldview in data analysis.^25^

Video-recordings of actual clinical interactions will be analysed using linguistic and conversation-analytic methods to identify the skills that constitute culturally capable communication. These recordings will serve as the input for the development of video-stimulated interviews^26^; wherein, consumers and mental health professionals will be interviewed, individually and separately, and shown the recording of their consultation to prompt a conversation with the interviewer about their subjective experience at specific moments during that clinical interaction.

The findings from these data analyses will be incorporated into the development of the training package (see the above section ‘Development of the communication training program’ for details); and quantitative/survey data collected from the HPAG will be analysed using inter-rater agreement and content validity index to determine whether the communication training programme and its measurement scales are relevant for the implementation phase. HPAG members may also provide open-ended feedback.

### Phase 2

For Phase 2, implementation of the communication training programme has not yet commenced. Based on current projections, Phase 2 is expected to begin in 2027 following completion of Phase 1 activities, finalisation of the training package, and receipt of all required governance and ethics approvals. The CTP will be delivered across the four sites using a staggered rollout, and data will be collected at pre- and post-training time intervals. Three frameworks were considered to guide the research team in formulating the evaluation model. At the mental health professional-level, we refer to the training evaluation model by Kirkpatrick and Kirkpatrick.^27^ This model guides the evaluation of mental health professionals in terms of (1) their reaction to or satisfaction with the training programme, (2) changes in their self-efficacy, knowledge, and attitudes regarding mental health communication and (3) changes in clinical practice. At the consumer-level, three themes from the Patient Reported Indicator Surveys^28^ were used to evaluate consumers’ (1) experience and (2) characteristics. Lastly, we will evaluate the impact at the broader health system-level by observing changes in attendance rate and total cost of service delivery as it is anticipated that increased attendance at mental healthcare appointments (post-intervention) in early stages of illness/distress will prevent progression of the illness to requiring costly hospital admissions.

Post-training measurement will occur at three time points: (1) immediately following, (2) 1 month after and (3) 6 months later. Pre- and post-training measurements will consist of multidimensional and multimodal assessments of communication between mental health professionals and consumers, using online surveys and consumers’ health records. Table 1 outlines all planned outcome measures.

#### Sample size

It is expected that 50% of non-Indigenous mental health professionals who provide direct care to Aboriginal and Torres Strait Islander mental health consumers at the four trial sites will participate in the communication training. Table 2 outlines their distribution across the HHSs. This proportion will serve as a reference point for determining the adequate sample size for statistical inference.

**Table 2:** Proportion of non-Indigenous mental health professionals across four HHSs

Study sites (HHSs)	Number of mental health professionals
Metro North	199
Metro South	202
Cairns and Hinterland	171
Central Queensland	130
Total	702

HHSs, Hospital and Health Services.

Determining the sample size for this study is challenging due to the study design’s complexity and the multidimensional, multimodal approach to assessment. Given the collaboration with Queensland Health, it is also expected that the communication training will involve all mental health professionals who meet the previously described inclusion criteria. To estimate the sample size, we assumed that participants’ positive attitudes towards communicating with consumers would improve following the training. A statistical power analysis using the ‘pwr’ package in R^29^ assumed a small effect size (d=0.30), a power of 0.80 and a significant level of 0.05. The minimum sample size required is 90 individuals at each study site. The anticipated lower participation rate in Central Queensland HHS, where the number of mental health professionals is lower than in the other HHSs, will be mitigated through the support of a local site coordinator and the Executive Director of Aboriginal and Torres Strait Islander Health and well-being, who will help ensure a high level of participation among all eligible participants. Additionally, multilevel modelling may be used to account for the nested structure of the data and the imbalance in sample sizes between HHSs. Random effects will be estimated where appropriate, with the expectation that the HHS with smaller sample sizes will have less precise estimates and wider CIs.

#### Outcome measures

This study protocol includes a list of outcomes and measures to evaluate the communication training programme (table 1). However, due to the co-design process, it is likely that the measures will be further adjusted based on the information acquired in Phase 1 and with co-design input from the CAG and HPAG. We also recognise the agile processes and the need to be sensitive and reflexive in the evolution of the training.

Training satisfaction will be assessed with the Training Acceptability Rating Scale TARS-2 which consists of nine items^30^ to measure the overall impression of the training programme rated on a four‐point Likert scale, ranging from ‘not at all’ (score 0) to ‘a great deal’ (score 3). Following the self-rating section, it concludes with three open-ended questions asking about the ‘most helpful’ part of the training, any ‘recommended changes’ and ‘any other comments’. The sum of the scores indicates the perceived impact of the training with higher scores representing higher levels of satisfaction with the training programme.

Mental health professionals’ self-efficacy in communication with their consumers will be assessed using the Self-Efficacy Questionnaire (SE-12), developed within the context of clinical communication skills for healthcare professionals.^31^ The scale comprises 12 items, each beginning with the words: ‘How certain are you that you are able to successfully…’ followed by a specific communication skill, such as ‘…make an agenda/plan for the conversation with the patient?’. Professionals provide responses on a 10-point scale ranging from 1 (very uncertain) to 10 (very certain). Various psychometric evidence, such as a Cronbach’s α of 0.95, shows that this scale can be used to measure clinicians’ self-efficacy in communication.^31^

Mental health professionals’ attitude towards communication with their consumers will be assessed with a 12-item unidimensional Attitude Towards Medical Communication Scale with Cronbach’s α of 0.74.^32^ An example of the item is ‘Good communication is a core clinical skill’. Responses will be given in a Likert scale from 1 (‘strongly disagree’) to 5 (‘strongly agree’).

We will also use service level data and health record data to explore impacts on practitioner and client behaviour and outcomes. To evaluate A5, inductive coding of clinical notes recorded in the Consumer Integrated Mental Health and Addiction application as the designated medical record in Queensland under the Mental Health Act 2016 will be undertaken. Additionally, data from consumers’ medical records, Queensland Hospital Admitted Patient Data Collection, Emergency Data Collection and State National Hospital Cost Data Collection will be used to explore changes in clients’ outcomes (A6), including mental health status, their planned attendance rate vs actual, and the number of unplanned mental health hospital admissions; data that is also relevant to service-related costs.

Some areas of evaluation will be developed following the training development phase. For example, changes in knowledge and practice will be assessed once the training package content has been finalised. Using the established strategies for developing and validating new scales,^33^ the knowledge and practice dimensions will first be conceptualised. The co-design process, such as yarning with the advisory group, will help determine the most appropriate measurement strategy to evaluate the impact of the communication training on mental health professionals’ knowledge. Two strategies are currently being considered: a self-assessment communication scale^34^ and a situational judgement test.^35^ These approaches have previously been used in medical and communication skills assessment contexts, respectively. Following conceptualisation, the item-generation process will commence, followed by content validation. The final assessment form will then be presented to the advisory group to ensure that the validated items align with the yarning previously undertaken.

For measurements at the consumer-level, we use the framework used in the Patient-Reported Indicator Survey.^28^ We will measure consumers’ experience using the patient-reported experience tool that is commonly used among Aboriginal and Torres Strait Islander consumers.^7^ Consumers will report their perception of the time that was given to them for consultation, of whether they were being treated with respect, and whether they were given the opportunity to ask questions.

Consumer characteristics encompass socio-demographic background, health behaviours and engagement with the service.^28^ In this study, we specifically focus on consumers’ engagement as reflected through their perceived therapeutic relationship. Changes in consumers’ engagement will be measured with the Kim Alliance Scale.^36^ The 16-item scale consists of four dimensions including collaboration, integration, empowerment and communication. An example item is: ‘I feel my provider does not listen to me’ (reverse-scored item). Consumers can provide answers ranging from never (1) to always (4).

In addition to standardised measures and in-house developed measures, we will also collect data that may explain changes in the outcome of the communication training. This data will include socio-demographic factors of the mental health professionals, such as age, education level, length of experience working in the field and occupational status.

#### Data analysis

Quantitative data obtained through surveys and medical records will be analysed using descriptive statistics and data visualisation frameworks. Mean, SD and box plots will describe sample characteristics, data distribution and proportionality. The phased implementation provides a setting for a staggered difference-in-differences (DiD) design, which estimates treatment effects by comparing observed outcomes with counterfactual outcomes across units exposed to the intervention at different times.^37^ By using the not-yet-treated group as the control, this approach mitigates the risk of bias from structural differences between treatment and comparison groups, as all units eventually receive the intervention at varying times. This design offers a robust and credible strategy for causal inference in estimating the effects of the communication training.^38^

To strengthen causal inference, outcomes derived from routinely collected clinical and administrative data will be converted into a longitudinal panel comprising repeated observations at 1-month intervals for each participating service. The communication training programme will be implemented sequentially across the four HHSs, creating variation in treatment timing that facilitates a staggered DiD design. At any given time point, services that have not yet received the training will serve as the comparison group for those already exposed to the intervention. Analyses will be conducted using individual-level repeated measures models that account for clustering within sites and serial correlation over time, with site and calendar-month fixed effects included to control for time-invariant differences between services and secular trends. Consistent with contemporary staggered DiD methods,^37^ treatment effects will be estimated using group–time average treatment effects, and the plausibility of the parallel trends assumption will be assessed through examination of pre-intervention outcome trajectories and event-study estimates. Although the staggered design supports examination of changes over time, the absence of randomisation means that group differences may have influenced the observed effects. Causal interpretations are therefore made cautiously.

### Ethics and concluding words

This protocol has described how the communication training will be co-designed, implemented and evaluated with a strong cultural consideration from the perspective of Aboriginal and Torres Strait Islander peoples. In addition, multidimensional evaluation strategies have been outlined to obtain robust findings across diverse outcomes. The primary outcome of this study is the improved knowledge of non-Indigenous mental health professionals in communicating with Aboriginal and Torres Strait Islander consumers. Observable changes in communication practices are expected, with mental health professionals incorporating more cultural information into their clinical notes and demonstrating greater cultural sensitivity with their Indigenous consumers.

The ethical considerations for this study are primarily guided by the AIATSIS Code of Ethics for Aboriginal and Torres Strait Islander Research,^39^ which addresses issues such as data sovereignty, data storage and risk mitigation. The Principal Investigator and the Steering Committee, which comprises Indigenous members of the research team, made decisions regarding how the data are stored and analysed. For example, sensitive data, such as audio and video recordings, will be stored in a secure, dedicated local research data storage system, with access restricted to the Project Coordinator in their role as the data custodian. They also determined how the study findings are published and returned to participating services, advisory groups and Aboriginal and Torres Strait Islander communities, while ensuring that all publications accurately reflect the experiences and knowledges of Indigenous peoples.

The study is also being conducted in collaboration with Queensland Health, which will provide support for the management of participant distress. For example, Indigenous consumer participation will be facilitated through nomination by a mental health professional to minimise the risks associated with approaching consumers who may be distressed or otherwise adversely affected by an invitation to participate in the study. Consumers will still be required to self-nominate before deciding whether to participate. Procedures to manage participant distress include the right to pause or withdraw from the study at any time, as well as access to appropriate support pathways should distress arise.

Phase 1 of the study was approved by the Darling Downs Health HREC on 26 September 2022 (HREC/2022/QTDD/81224). To date, only the data collection involving yarning with mental health professionals has been conducted. Other aspects of the Phase 1 data collection have not yet been carried out. Following the conclusion of Phase 1, separate ethics approval for Phase 2 will be sought.

It is expected that there will be an increase in positive attitudes and greater self-efficacy of non-Indigenous mental health professionals in communicating with Aboriginal and Torres Strait Islander consumers (secondary outcomes). In addition, it is expected that higher consumer satisfaction with care received compared with consumer satisfaction prior to their mental health professionals attending the training will be measured.

We will explore the impact of CTP on consumer engagement with services as a consequence of mental health provider attendance at the training; ideally, reviewing attendance rates at scheduled appointments generally and engagement with services for the 7-day period following an acute episode and/or admission to hospital; comparing engagement and attendance prior to their mental health professional attending the training. Outcomes will be explored via a staggered training rollout across the four trial sites and using a quasi-experimental, DiD framework. The main benefit of using a DiD is the ability to estimate causal effects using observational data, controlling for both fixed-group and time-varying effects.^40^ In this way, we can identify any effects due to training, minimising the likelihood of other factors (eg, non-Indigenous mental health professionals’ access to digital resources) confounding analysis and findings.

To reduce the risk of harm for Aboriginal and Torres Strait Islander participants, we have employed specific strategies across the lifecycle of the project. First, the co-design approach was chosen to assist the research team in engaging with community members and ensuring that the study design is respectful and fair to the involved communities.^41^ The research is led by a team comprising a majority of Aboriginal and/or Torres Strait Islander investigators. The team are compliant with, advocate for and demonstrate a significant track record as experts in the conduct of research that adheres to and respects local, jurisdictional, national and internationally accepted best practice in the conduct of research involving Aboriginal and/or Torres Strait Islander peoples.

A single-arm intervention study was chosen to mitigate the negative impact due to unforeseen consequences of the trial. A single-arm study may have limitations due to the lack of a comparison with a control group. Consequently, the conclusions may also be influenced by other factors occurring around the same time as the intervention.^42^ To address this limitation, the study will also use a DiD approach to examine changes in outcomes before and after the training rollout. Furthermore, many pilot studies use single-arm trials to collect more data before fully launching a randomised controlled trial,^43 44^ demonstrating the potential benefits of single-arm interventional studies. Finally, the communication training programme will be developed specifically for this study. Therefore, the single-arm design may reduce potential harm to Aboriginal and/or Torres Strait Islander participants, as the current evidence does not guarantee a low level of risk of the communication training programme.

A key element of this study is the incorporation of cultural sensitivity into both research and clinical practices. Our commitment to cultural sensitivity is reflected through the inclusion of four sites, each representing different Aboriginal and Torres Strait Islander communities. Aboriginal and Torres Strait Islander communities are numerous and diverse, representing more than 200 different language groups, with distinct cultural practices, traditions, languages, values, familial and social systems. Further, family, community, country and place were found to be crucial factors that shape the identity of Indigenous peoples.^45^ Mayi Kuwayu, a national study aiming to develop a culturally sensitive health and well-being survey tool, considered the representation of locations of the participating communities, spanning across saltwater, freshwater, desert, island groups, urban, regional and remote areas.^46^ This prior study also considered whether the communities experienced specific life events, such as forced removals.^46^ All these considerations were included to ensure that the findings would be applicable and meaningful across a range of Aboriginal and Torres Strait Islander communities. Reflecting on these considerations, we acknowledge the importance of location where the study takes place and full representation of Aboriginal and Torres Strait Islander communities. This study has limitations in terms of generalisability of its findings, and we should attempt to reach further communities in various locations. At this point, the current study will serve as a scaffold to build knowledge about the importance of education in communication training for mental health professionals.
