# Supplementary material for: Implementing a co-designed communication training programme for non-Indigenous mental health professionals working with Aboriginal and/or Torres Strait Islander consumers in Queensland, Australia: protocol
Source: BMJ Open. 2026 Oct 29;16(10):e117447. doi: 10.1136/bmjopen-2026-117447 (PMC13723805; doi:10.1136/bmjopen-2026-117447)
Supplement: Supplementary file 1 [file bmjopen-2026-117447.reviewer_comments.pdf]

PEER REVIEW HISTORY

BMJ Open publishes all reviews undertaken for accepted manuscripts. Reviewers are asked to complete a checklist review form and are provided with free text boxes to elaborate on their assessment. These free text comments are reproduced below.

ARTICLE DETAILS

Title (Provisional)

PROTOCOL: Implementing a Co-Designed Communication Training Program for Non-Indigenous Mental Health Professionals Working with Aboriginal and/or Torres Strait Islander Consumers in Queensland, Australia

Authors

Surijah, Edwin Adrianta; Bernardes, Christina M; Ekberg, Stuart; Rogers, Julie-Anne; Spinks, Jean Marie; Meuter, Renata; Burke, Kylie; Simcock, Gabrielle Paton; Combo, Michelle; Mushin, Ilana; Pratt, Greg

---

VERSION 1 - REVIEW

**Reviewer**	**1**
**Name**	**Wu, Dadong**
**Affiliation**	**UCL Institute for Global Health**
**Date**	**02-Jun-2026**
**COI**	**None**

This protocol addresses an important and highly relevant topic: the development and evaluation of a culturally sensitive communication training programme for non-Indigenous mental health professionals working with Aboriginal and/or Torres Strait Islander consumers. The proposed study is strengthened by its co-design approach, First Nations governance structure, use of yarning as a culturally appropriate method, and its attempt to evaluate outcomes at professional, consumer and health-system levels.

However, I think several important aspects of the protocol require clarification before publication.

1. The study design needs clearer description. The manuscript describes the study as a mixed-methods, co-designed, single-arm interventional study with staggered rollout and a matched-sample observational component. The authors should clarify how these components relate to each other and whether the evaluation is primarily a single-arm pre–post study, a staggered quasi-experimental study, or a matched observational study.

2. The study timeline should be specified more clearly. As this is a protocol, the manuscript should state the planned or actual dates for Phase 1 data collection, training development, Phase 2 implementation, follow-up assessments, and study completion. It should also clarify which parts of Phase 1 have already been completed.

3. Ethics information requires clarification. The manuscript states that Phase 1 was approved in 2022 and that separate ethics approval for Phase 2 will be sought. The authors should clearly state which ethics committee approved which phase, the approval number(s), and how the study will proceed if Phase 2 approval is still pending.

4. The primary outcome and measurement strategy should be defined more precisely. Improved knowledge among non-Indigenous mental health professionals is later described as the primary outcome, but the knowledge measure has not yet been developed. The authors should clarify how the primary outcome will be measured and at which time point.

5. The follow-up time points need correction and clarification. The text mentions measurements immediately after training, one month after training, and six months later, but Table 2 lists t0, t1 and t2, while the note refers to t3. These inconsistencies should be corrected.

6. The sample size and analysis plan need further justification. The authors report that 90 participants are required at each site, but expected participation may be lower in some sites. They should explain how lower recruitment will be handled. The proposed difference-in-differences analysis also requires more detail on rollout timing, comparison groups, clustering by site, repeated measures, and key assumptions.

7. Recruitment and data governance need more detail. Consumers will be nominated by mental health professionals, which may introduce selection bias or gatekeeping. The authors should explain how this will be mitigated. They should also provide more detail on data ownership, data sovereignty, management of sensitive audio/video recordings, participant distress protocols, and how findings will be returned to participating communities.

Minor comments

1. The terminology used for “Indigenous,” “First Nations,” and “Aboriginal and/or Torres Strait Islander” should be standardized as much as possible, while respecting community preferences.

2. The abstract should avoid presenting expected findings as “Results.” For a protocol, this section could be reframed as “Expected outcomes” or incorporated into the conclusion.

3. The authors should consider whether a protocol registration number is required or appropriate, given that Phase 2 involves an interventional evaluation.

4. The data analysis section should specify how qualitative findings from yarning, video-stimulated interviews and conversation analysis will be integrated into the final training design.

5. The manuscript would benefit from some language tightening, particularly in sections where the rationale and governance arrangements are repeated.

**Reviewer**	**2**
**Name**	**Fonseca, Laura**
**Affiliation**	**Universidad de La Sabana**
**Date**	**03-Jun-2026**
**COI**	**None**

Thank you for the opportunity to review this article. This is an important study examining a relevant and interesting topic. The study will be enhanced if the framing and methodological approach are better contextualised. Particularly, it will benefit if the authors improve the introduction with a wider framework and provide specific details on the programme's evaluation. I have provided major comments below.

1. It is important to provide an initial paragraph that locates the study at the global level. Why is co-design important? What are other studies that include lived experience users in other countries with similar populations? Page 6 line 7, you mention previous studies but there are no specific references.

2. In the section "Development of the Communication Training Programme", even though you define yarning, a more specific definition would be useful for readers. For example, how does the relationship-building occur? How does the creation of new knowledge happen? I think this is a great methodological tool that can inform other research processes and needs more detail. The same thing applies to how it will be conducted in relation to local customs and sensitivities. More detail is needed here to understand the rationale of the implementation of yarning.

3. In phase 2, it is not clear why the total cost of service delivery is an indicator of improvement in services. Could you elaborate on this?

4. I would like to know more about the role of participants in phase 2 and onwards. Are they going to be involved in the evaluation and data analysis? If this is a PAR project, their involvement and active participation are crucial.

5. In the concluding words section, you refer to expected outcomes (primary and secondary). Even though you mention them in the methodology section, it would be useful to have a more specific table that includes outcome evaluation parameters and another with outcome evaluation measures that includes the specific outcome, the indicator and the measures. This can inform the concluding words, where you mention specifically the primary and secondary outcomes of the study.

VERSION 1 - AUTHOR RESPONSE

We would like to thank the two reviewers for their positive feedback regarding the importance and relevance of this topic, and for their time critiquing the manuscript. We sincerely appreciate their insightful and constructive feedback, which has helped us to improve our manuscript. In the following matrix, we address each comment and suggestion provided by the editor and reviewers.

No.	Comment/Feedback	Response
**Reviewer A Comments**		
This protocol addresses an important and highly relevant topic: the development and evaluation of a culturally sensitive communication training programme for non-Indigenous mental health professionals working with Aboriginal and/or Torres Strait Islander consumers. The proposed study is strengthened by its co-design approach, First Nations governance structure, use of yarning as a culturally appropriate method, and its attempt to evaluate outcomes at professional, consumer and health-system levels. However, I think several important aspects of the protocol require clarification before publication.		
RA1	The study design needs clearer description. The manuscript describes the study as a mixed-methods, co-designed, single-arm interventional study with staggered rollout and a matched-sample observational component. The authors should clarify how these components relate to each other and whether the evaluation is primarily a single-arm pre–post study, a staggered quasi-experimental study, or a matched observational study.	Thank you for this feedback. We attempt to clarify this further by making this adjustment:<br><br>This is a mixed-methods, co-designed, single-arm interventional study evaluating a communication training program for mental health clinicians. The study comprises two phases (Figure 1). **Phase 1** will focus on the engagement and co-design of the communication training package with mental health professionals and consumers.<br>**Phase 2** will involve the implementation and evaluation of the training program. The evaluation employs a single-arm, non-randomised pre–post intervention design, with the training program rolled out sequentially across participating services in a staggered manner (Simon, et al., 2025). Outcomes will be assessed before and after training to examine changes associated with the intervention. To complement the primary pre–post evaluation, matched-sample observations derived from clinical health records will be analysed to provide additional evidence regarding the impact of the training program. These observational analyses serve as a supplementary component of the evaluation rather than a separate primary study design.
RA2	The study timeline should be specified more clearly. As this is a protocol, the manuscript should state the planned or actual dates for Phase 1 data collection, training development, Phase 2 implementation, follow-up assessments, and study completion. It should also clarify which parts of Phase 1 have already been completed.	Thank you for this suggestion. We agree that greater clarity regarding the study timeline is important and have revised the manuscript accordingly. We have addressed this feedback by adding the following information. After Figure 1, we have included additional details regarding Phase 1:<br>Based on Figure 1, Phase 1 has been partially completed, with the yarning circles involving non-Indigenous mental health professionals and Aboriginal and Torres Strait Islander mental

		health workers conducted between 18 November 2022 and 8 November 2024. Individual yarn-ups with consumers commenced on 16 June 2025 and are still ongoing. The recording of clinical consultations is planned for 2026.<br><br>For Phase 2, we have added more information as follows:<br>For Phase 2, we have added that implementation of the communication training program has not yet commenced. Based on current projections, Phase 2 is expected to begin in 2027 following completion of Phase 1 activities, finalisation of the training package, and receipt of all required governance and ethics approvals.<br><br>As this is a co-designed study conducted in partnership with Aboriginal and Torres Strait Islander stakeholders and health services, future milestones are necessarily dependent on ongoing community consultation, advisory group input, service readiness, and regulatory approvals. Consequently, we have reported projected rather than fixed dates for the remaining study activities and have clarified this in the manuscript.
RA3	Ethics information requires clarification. The manuscript states that Phase 1 was approved in 2022 and that separate ethics approval for Phase 2 will be sought. The authors should clearly state which ethics committee approved which phase, the approval number(s), and how the study will proceed if Phase 2 approval is still pending.	Thank you for this feedback. We have amended the ethics section as follows:<br><br>Phase 1 of the study was approved by the Darling Downs Health HREC ~~ethics committee~~ on 26 September 2022 (HREC/2022/QTDD/81224).
RA4	The primary outcome and measurement strategy should be defined more precisely. Improved knowledge among non-Indigenous mental health professionals is later described as the primary outcome, but the knowledge measure has not yet been developed. The authors should clarify how the primary outcome will be measured and at which time point.	Thank you for this crucial feedback. The primary outcome is change in participants' knowledge of culturally safe communication with Aboriginal and Torres Strait Islander consumers. This outcome will be assessed using a purpose-developed questionnaire co-designed during Phase 1. The questionnaire will be administered immediately before and immediately after training. The final domains and items will be informed by stakeholder consultation, including Aboriginal and Torres Strait Islander consumers, mental health professionals, and relevant literature.
RA5	The follow-up time points need correction and clarification. The text mentions measurements immediately after training, one month after training,	Thank you for this detailed feedback. We have corrected the inconsistency. We also clarify that ‘t2’ refers to the follow-up assessments

	and six months later, but Table 2 lists t0, t1 and t2, while the note refers to t3. These inconsistencies should be corrected.	conducted one month and six months after the training. The note for Table 2 now reads:<br><br>Note: t0 = baseline; t1 = post-intervention; t2 = follow-up one month and six months after training/intervention.
RA6	The sample size and analysis plan need further justification. The authors report that 90 participants are required at each site, but expected participation may be lower in some sites. They should explain how lower recruitment will be handled.<br><br>The proposed difference-in-differences analysis also requires more detail on rollout timing, comparison groups, clustering by site, repeated measures, and key assumptions.	Thank you for this feedback. Regarding the mitigation of low participation rate, we have added this information:<br>The anticipated lower participation rate in Central Queensland HHS, where the number of mental health professionals is lower than in the other HHSs, will be mitigated through the support of a local site coordinator and the Executive Director of Aboriginal and Torres Strait Islander Health and Wellbeing, who will help ensure a high level of participation among all eligible participants.<br><br>Regarding the DiD analysis, we have added further information as follow:<br>To strengthen causal inference, outcomes derived from routinely collected clinical and administrative data will be converted into a longitudinal panel comprising repeated observations at one-month intervals for each participating service. The communication training program will be implemented sequentially across the four Hospital and Health Services, creating variation in treatment timing that facilitates a staggered difference-in-differences design. At any given time point, services that have not yet received the training will serve as the comparison group for those already exposed to the intervention. Analyses will be conducted using individual-level repeated measures models that account for clustering within sites and serial correlation over time, with site and calendar-month fixed effects included to control for time-invariant differences between services and secular trends. Consistent with contemporary staggered DiD methods (Callaway & Sant'Anna, 2021), treatment effects will be estimated using group–time average treatment effects, and the plausibility of the parallel trends assumption will be assessed through examination of pre-intervention outcome trajectories and event-study estimates.
RA7	Recruitment and data governance need more detail. Consumers will be nominated by mental health professionals, which may introduce selection bias or gatekeeping. The authors should explain how this will be mitigated. They should also provide more detail on data ownership, data	Thank you for highlighting these important considerations. We have revised the manuscript to provide additional detail regarding recruitment procedures, data governance, participant welfare, and dissemination of findings. Please find our revision on section 'Ethics and Concluding Words'.

	sovereignty, management of sensitive audio/video recordings, participant distress protocols, and how findings will be returned to participating communities.	We acknowledge that clinician nomination of consumers may introduce selection bias or gatekeeping. This approach was adopted to ensure that potential participants are clinically appropriate for contact and able to provide informed consent. To mitigate this risk, clinicians will be provided with clear eligibility criteria and asked to identify all eligible consumers. We have also acknowledged the potential for residual selection bias as a study limitation.<br><br>We have expanded the data governance section to clarify that data management will be guided by Indigenous data sovereignty principles, primarily guided by the AIATSIS Code of Ethics for Aboriginal and Torres Strait Islander Research (Australian Institute of Aboriginal and Torres Strait Islander Studies, 2020), relevant ethics approvals, and project governance arrangements. Audio and video recordings will be stored securely on password-protected institutional servers with access restricted to authorised members of the research team.<br><br>We have also clarified participant distress procedures, including participants' right to pause or withdraw at any time and the availability of appropriate support pathways if distress arises.<br><br>Finally, we have added information that findings will be returned to participating services, advisory groups, and Aboriginal and Torres Strait Islander communities through culturally appropriate feedback processes.
RA8	The terminology used for “Indigenous,” “First Nations,” and “Aboriginal and/or Torres Strait Islander” should be standardized as much as possible, while respecting community preferences.	Thank you for this important feedback. We have amended the document and prioritised the term “Indigenous” for improved readability while also acknowledging the significance of the terminology “Aboriginal and Torres Strait Islander people” in the document and using “First Nations” when referring to First Nations peoples across continents.
RA9	The abstract should avoid presenting expected findings as “Results.” For a protocol, this section could be reframed	Thank you for this suggestion. We have made adjustments to the Abstract.

	as "Expected outcomes" or incorporated into the conclusion.	
RA10	The authors should consider whether a protocol registration number is required or appropriate, given that Phase 2 involves an interventional evaluation.	Thank you for this suggestion. We considered the appropriateness of prospective trial registration for this study. Although Phase 2 includes an interventional evaluation component, the present protocol primarily describes a co-design process through which the communication training program, its content, and aspects of its evaluation framework are being developed in partnership with stakeholders.<br>At this stage, the intervention has not yet been finalised, and key elements of the evaluation, including the specific knowledge domains and associated measurement tools, remain subject to the co-design process. As a result, the study does not yet have the level of intervention and outcome specification typically required for prospective trial registration.
RA11	The data analysis section should specify how qualitative findings from yarning, video-stimulated interviews and conversation analysis will be integrated into the final training design.	Thank you for this feedback. We have provided details on how the thematic findings from the Data Analysis will be incorporated into the training package into the section titled Development of the communication training program (see page 11). We have now included a statement to this effect in the Data Analysis section:<br><br>The findings from these data analyses will be incorporated into the development of the training package (see above section "Development of the communication training program") for details); and quantitative/survey data collected from the HPAG will be analysed using inter-rater agreement and content validity index to determine whether the communication training program and its measurement scales are relevant for the implementation phase. HPAG members may also provide open-ended feedback.
RA12	The manuscript would benefit from some language tightening, particularly in sections where the rationale and governance arrangements are repeated.	Thank you for this feedback. We have tightened up the wording for the study aims/rational and repetition regarding governance structure, as suggested.<br><br>For example:<br>~~The overall aim of this study is to further develop the communication skills of non-Indigenous health professionals working in mental health and to empower clinicians to reflect on how their ways of thinking, being, and doing influence their worldview of the mental health and wellbeing of~~

		~~Indigenous peoples.~~ We also set specific aims for each phase as follows:
**Reviewer B Comments**		
Thank you for the opportunity to review this article. This is an important study examining a relevant and interesting topic. The study will be enhanced if the framing and methodological approach are better contextualised. Particularly, it will benefit if the authors improve the introduction with a wider framework and provide specific details on the programme's evaluation. I have provided major comments below.		
RB1	It is important to provide an initial paragraph that locates the study at the global level. Why is co-design important? What are other studies that include lived experience users in other countries with similar populations?<br><br>Page 6 line 7, you mention previous studies but there are no specific references.	We have now included a broader introductory paragraph to provide broader /international context for the research topic and research methodology.<br><br>Effective communication between clinicians and First Nations patients/consumers underpins the delivery of high-quality, culturally safe, and equitable healthcare (Amery, 2017; Logan, Steel, & Hunt, 2014). Beyond the ongoing impacts of colonisation experienced by First Nations peoples, effective communication is particularly critical in mental healthcare, where clinician–patient communication serves as a primary diagnostic and therapeutic tool (Roman, et al., 2026). Communication barriers are also recognised as major contributors to disparities in mental health outcomes among First Nations consumers (Logan, Steel, & Hunt, 2014).<br><br>We also have now included int-text citations to support the comment about previous research.<br><br>Previous studies have shown the positive effects of co-designed communication training for non-Indigenous clinicians on other aspects of Indigenous healthcare (Bernardes, et al., 2022; Cass, et al., 2002; Kerrigan, et al., 2025; Wilson, et al., 2020).
RB2	In the section "Development of the Communication Training Programme", even though you define yarning, a more specific definition would be useful for readers. For example, how does the relationship-building occur? How does the creation of new knowledge happen? I think this is a great methodological tool that can inform other research processes and needs more detail. The same thing applies to how it will be conducted in relation to local customs and sensitivities. More detail is needed here to understand the rationale of the implementation of yarning.	We have now expanded our explanation of the Yarning methods in this section to address the methodological details requested (pg 12-13):<br>Relationship building will occur through the establishment of culturally safe spaces, which use appropriate introduction and informal conversations, the recognition of local cultural protocols (e.g. Acknowledgment of Country, Elders, key staff) and the facilitation of discussions by researchers experienced in yarning. Participants will discuss issues they consider important to communication within mental health services, allowing priorities and insights to emerge organically. This enables the generation of new knowledge, creating opportunities for reflection and identification of shared strengths and solutions. At each study site,

		yarning will be adapted to local customs and community expectations.<br>Guidance from local Aboriginal and Torres Strait Islander mental health professionals will inform decisions regarding participant recruitment, the composition of yarning groups, appropriate venues, facilitation processes, and the management of culturally sensitive topics. This flexibility recognises the diversity of Aboriginal and Torres Strait Islander communities and ensures that the research process remains locally relevant, respectful and culturally responsive. The yarning circles will also explore mental health professionals' preferences for the communication training program (eg, duration, mode of delivery, training resources).<br><br>We have improved the rational for using yarning in the Introduction (pg 5):<br>Understanding what constitutes culturally appropriate communication with Aboriginal and Torres Strait Islander consumers requires the use of culturally appropriate co-design methodology (eg, yarning) (Bessarab & Ng'andu; 2010). Indigenous research methods provide culturally safe and relational ways of gathering knowledge that may not be accessible through conventional data collection approaches. By generating evidence that is grounded in Indigenous worldviews and lived experiences, these methods can better inform service design, delivery, and quality improvement initiatives, contributing to meaningful service-level change and improved outcomes. Yarning is more than a conversational data collection technique; it's a culturally grounded process that builds trust and respectful relationships through shared dialogue, active listening and the recognition of participants as knowledge holders. The yarning process allows participants to share experiences, stories and reflections in ways that are culturally safe and meaningful, enabling knowledge to emerge collaboratively rather than being extracted by researchers.
RB3	In phase 2, it is not clear why the total cost of service delivery is an indicator of improvement in services. Could you elaborate on this?	We have now elaborated on why cost effectiveness of service delivery will be an indicator of improved services:<br><br>Lastly, we will evaluate the impact at the broader health system-level by observing changes in attendance rate and total cost of service delivery as it is anticipated that increased attendance at mental health care appointments (post-intervention) in early stages of illness/distress will prevent progression of the illness to requiring costly hospital admissions.

RB4	I would like to know more about the role of participants in phase 2 and onwards. Are they going to be involved in the evaluation and data analysis? If this is a PAR project, their involvement and active participation are crucial.	We have now included the ongoing involvement of participants in Phase 2 of the Study (pg 13):<br>Findings from the yarning process will inform the development of a draft communication training program, which will subsequently be subjected to iterative cycles of affirmation, refinement and confirmation workshops in conjunction with the participants who wish to join the Consumer Advisory Group in Phase 2 of the study.<br><br>And further elaboration of involvement in Phase 2 evaluation Methods (pg 16-17):<br><br>However, due to the co-design process, it is likely that the measures will be further adjusted based on the information acquired in Phase 1 and with co-design input from the CAG and HPAG.
RB5	In the concluding words section, you refer to expected outcomes (primary and secondary). Even though you mention them in the methodology section, it would be useful to have a more specific table that includes outcome evaluation parameters and another with outcome evaluation measures that includes the specific outcome, the indicator and the measures. This can inform the concluding words, where you mention specifically the primary and secondary outcomes of the study.	Thank you for this point. We have now expanded Table 2 to include the level of outcome (primary or secondary) to compliment the statements in the Concluding Words section. See Table 2, page 15.

VERSION 2 - REVIEW

**Reviewer**	**1**
**Name**	**Wu, Dadong**
**Affiliation**	**UCL Institute for Global Health**
**Date**	**29-Jul-2026**
**COI**	

The authors have substantially improved the protocol. The study design, timeline, Indigenous governance, ethics and data sovereignty arrangements, and staggered evaluation plan are now much clearer. Most of my previous concerns have been adequately addressed.

I have only a few remaining minor comments:

The primary outcome is now defined as improved knowledge among non-Indigenous mental health professionals, but the knowledge measure has not yet been developed. Please briefly clarify how this measure will be developed, scored, and assessed for content validity.

The follow-up time points should be presented more clearly. The text refers to immediate post-training, one-month follow-up, and six-month follow-up, but Table 2 combines the latter two under t2. Please revise the table to avoid confusion.

The sample size section should clarify how the study will handle sites where recruitment may fall below the target of 90 participants, particularly Central Queensland.

If a "matched-sample observational" component remains part of the design, please briefly describe the matching procedure. If not, please revise the terminology to align with the staggered difference-in-differences evaluation.

Please use cautious language when describing causal inference from the non-randomised staggered design.

Overall, the revised manuscript is much clearer and stronger. I recommend minor revision.

VERSION 2 - AUTHOR RESPONSE

Thank you for the latest feedback to our manuscript ID bmjopen-2026-117447. In the following matrix, we address each comment and suggestion provided by the editor and reviewers.

No.	Comment/Feedback	Response
**Reviewer A Comments**		
RA1	The primary outcome is now defined as improved knowledge among non-Indigenous mental health professionals, but the knowledge measure has not yet been developed. Please briefly clarify how this measure will be developed, scored, and assessed for content validity.	We have added a brief remark on this planned process:<br><br>Some areas of evaluation will be developed following the training development phase. For example, changes in knowledge and practice will be assessed once the training package content has been finalised. Using the established strategies for developing and validating new scales (33), the knowledge and practice dimensions will first be conceptualised. The co-design process, such as yarning with the advisory group, will help determine the most appropriate measurement strategy to evaluate the impact of the communication training on mental health professionals' knowledge. Two strategies are currently being considered: a self-assessment communication scale (34) and a situational judgement test (35). These approaches have previously been used in medical and communication skills assessment contexts, respectively. Following conceptualisation, the item-generation process will commence, followed by content validation. The final assessment form will then be presented to the advisory group to

		ensure that the validated items align with the yarning previously undertaken.
RA2	The follow-up time points should be presented more clearly. The text refers to immediate post-training, one-month follow-up, and six-month follow-up, but Table 2 combines the latter two under t2. Please revise the table to avoid confusion.	We have changed 't2' with 'f' and the note at the bottom of the table describes:<br><br>Note: t0 = baseline; t1 = post-intervention; f = follow-up one month and six months after training/intervention.
RA3	The sample size section should clarify how the study will handle sites where recruitment may fall below the target of 90 participants, particularly Central Queensland.	We have added this statement:<br><br>The anticipated lower participation rate in Central Queensland HHS, where the number of mental health professionals is lower than in the other HHSs, will be mitigated through the support of a local site coordinator and the Executive Director of Aboriginal and Torres Strait Islander Health and Wellbeing, who will help ensure a high level of participation among all eligible participants. Additionally, multilevel modelling may be used to account for the nested structure of the data and the imbalance in sample sizes between HHSs. Random effects will be estimated where appropriate, with the expectation that the HHS with smaller sample sizes will have less precise estimates and wider confidence intervals.
RA4	If a "matched-sample observational" component remains part of the design, please briefly describe the matching procedure. If not, please revise the terminology to align with the staggered difference-in-differences evaluation.	We have added a brief description:<br><br>Outcomes will be assessed before and after training to examine changes associated with the intervention. To complement the primary pre–post evaluation, matched-sample observations derived from clinical health records will be analysed to provide additional evidence regarding the impact of the training program. The matched-sample study refers to data requested from medical records, in which consumers' data will be matched with those of their treating mental health professionals. Changes in consumers' service access rates, health outcomes, and other relevant indices will be examined before and after the communication training attended by mental health professionals. These observational analyses serve as a supplementary component of the evaluation rather than a separate primary study design.
RA5	Please use cautious language when describing causal inference from the non-randomised staggered design.	We have added this statement to the data analysis section, which refers to the causal statement:<br><br>Although the staggered design supports examination of changes over time, the absence of randomisation means that group differences may have influenced the observed effects. Causal interpretations are therefore made cautiously.
